# Rewiring KRAS-driven cancers through the ubiquitin–proteasome system: therapeutic opportunities with a focus on deubiquitinase

**DOI:** 10.1038/s12276-026-01837-6

**Published:** 2026-09-07

**Authors:** Yeonjoo Lee, SuA Hwang, Hyungkyung Shin, Kyeongeun Choi, SeungYeon Seo, HyoKyoung Kim, Ji Su Yang, Yeon Jung Kim, Young-Mee Kim, Eun Joo Song

**Affiliations:** 1 Graduate School of Pharmaceutical Sciences and College of Pharmacy, Ewha Womans University, Seoul, Republic of Korea; 2 Graduate Program in Innovative Biomaterials Convergence, Ewha Womans University, Seoul, Republic of Korea; 3 Department of Biochemistry and Molecular Genetics, University of Illinois Chicago, Chicago, IL USA

**Keywords:** Ubiquitylation, Oncogenes

## Abstract

KRAS is one of the most frequently mutated oncogenes in human cancers and plays a central role in regulating signaling pathways that control cell proliferation, survival and metabolism. Recent advances in allele-specific inhibitors, particularly those targeting KRAS^G12C^, have demonstrated that direct pharmacological inhibition of KRAS is clinically feasible. However, the durability of therapeutic responses remains limited due to intrinsic and acquired resistance mechanisms, including secondary KRAS mutations, pathway reactivation and adaptive signaling rewiring. These challenges highlight the need for complementary strategies that extend beyond direct catalytic inhibition. The ubiquitin–proteasome system has emerged as a critical regulator of KRAS stability and signaling networks. Deubiquitinases (DUBs), which reverse ubiquitination and modulate protein turnover, have been increasingly recognized as key regulators of oncogenic signaling pathways upstream and downstream of KRAS. By influencing protein abundance, localization and signaling competence, DUBs can modulate pathway robustness and adaptive responses that contribute to therapeutic resistance. In this Review, we summarize current understanding of ubiquitin-dependent regulation in KRAS-driven cancers, focusing on DUBs and E3 ligases that directly control KRAS stability as well as those that regulate major KRAS effector pathways, including RTK–RAS, RAF–MEK–ERK and PI3K–AKT–mTOR signaling. We further discuss the therapeutic implications of DUB inhibition, its potential to overcome resistance and its relationship to emerging targeted protein degradation strategies.

## Overcoming KRAS resistance through ubiquitin system insights

KRAS, a small GTPase, plays a pivotal role in cell growth and survival, and its mutations are prevalent in various cancers, including pancreatic, colorectal and lung cancers. This Review explores the development of KRAS inhibitors, highlighting the challenges due to KRAS’s ‘undruggable’ nature and the success of covalent inhibitors such as sotorasib for KRAS^G12C^ mutant cancers. The authors discuss the limitations of these inhibitors, such as resistance mechanisms that involve secondary mutations and pathway reactivation and summarize studies describing these processes. Importantly, the Review highlights the ubiquitin–proteasome system, particularly deubiquitinases, as key regulators of KRAS stability and signaling networks, providing complementary therapeutic opportunities beyond direct KRAS inhibition. The findings underscore the need for protein-level regulation and combination strategies to overcome resistance and improve the durability and efficacy of KRAS-targeted therapies.

This summary was initially drafted using artificial intelligence, then revised and fact-checked by the author.

## Introduction

### Biological functions of KRAS in physiological conditions and its role in cancer

KRAS is a small GTPase that serves as a central signaling hub regulating cell growth, survival and differentiation^1^. In physiological settings, KRAS activation is initiated by upstream receptor tyrosine kinases (RTKs) that engage guanine nucleotide exchange factors (GEFs), promoting GDP-to-GTP exchange and generating the active, GTP-bound state. Activated KRAS propagates signals through multiple effector pathways, most prominently the RAF–MEK–ERK^2^ and PI3K–AKT–mTOR cascades, as well as additional networks that collectively support proliferation, survival and metabolic adaptation^2^. Accordingly, dysregulated KRAS activity profoundly affects tumorigenic signaling and cancer progression.

Oncogenic KRAS mutations occur across diverse tumor types and exhibit substantial allele- and context-specific heterogeneity. Frequent hotspot mutations—including G12C, G12D, G13D and Q61H—impair GTP hydrolysis and bias KRAS toward a constitutively active state^3,4^. The prevalence of KRAS mutations is highly cancer specific, with particularly high frequencies in pancreatic, colorectal, and lung cancers^4^. Less common variants, such as A146T and K117N, can also promote aberrant KRAS activation. Regardless of mutational subtype, most KRAS oncogenic variants drive persistent downstream signaling, establishing KRAS as a long-standing and compelling therapeutic target.

### Development of KRAS inhibitors and their limitations

Despite its clinical relevance, direct pharmacological targeting of KRAS was historically challenging. KRAS presents limited druggable pockets, binds guanine nucleotides with high affinity in a nucleotide-rich intracellular environment, and undergoes conformational transitions between the GDP- and GTP-bound states, which complicate ligand design. Accordingly, KRAS was widely regarded as an ‘undruggable’ oncoprotein for decades^5^.

A breakthrough was achieved with covalent inhibitors targeting KRAS^G12C^, exemplified by sotorasib and adagrasib^6^. These agents covalently engage the mutant cysteine residue and trap KRAS^G12C^ in the inactive GDP-bound conformation, enabling clinically meaningful responses, particularly in non-small cell lung cancer (NSCLC)^7^. This success accelerated the development of next-generation KRAS^G12C^ inhibitors, including D-1553 (garsorasib)^8^, JDQ443 (opnurasib), IBI351 (fulzerasib), JAB-21822 (glecirasib) and GDC-6036 (divarasib)^9^, which aim to improve pharmacokinetic properties, target engagement and mutant selectivity.

By contrast, KRAS^G12D^—prevalent in pancreatic and colorectal cancers—lacks a reactive cysteine, making covalent targeting more challenging than for KRAS^G12C^ and requiring the development of selective noncovalent inhibitors^10^. Several KRAS^G12D^-selective inhibitors have entered clinical development^11–13^. In addition, pan-KRAS inhibitors^14–16^ and pathway-level strategies targeting upstream regulators, such as SOS1 inhibitors^17^, are also being developed to achieve broad therapeutic coverage across diverse KRAS-mutant cancers (Supplementary Table 1).

Despite these advances, the durability of responses to KRAS inhibitors remains limited. Both intrinsic and acquired resistance mechanisms have been increasingly recognized, underscoring the adaptive capacity of KRAS-driven signaling networks and motivating the development of complementary strategies to improve response depth and duration^13^.

### KRAS inhibitor resistance mechanism and complementary strategies

Resistance to KRAS-targeted therapies arises through multiple mechanisms that can be broadly categorized as: (1) on-target secondary alterations in KRAS, (2) reactivation of downstream or parallel signaling pathways and (3) co-occurring genomic alterations that reshape pathway dependencies^13,18^. Genomic analyses revealed that a subset of adagrasib-resistant patients (approximately 18%) developed secondary KRAS alterations at residues G12, G13, Q61, H95 and Y96, which are known to impair inhibitor binding. More broadly, resistance was frequently driven by pathway reactivation through MET amplification (an RTK that activates RAS–MAPK signaling), activating mutations in RAS–MAPK components, alterations in NF1 or PTEN, and oncogenic fusions^19^.

Consistent with these findings, resistance to sotorasib frequently arises through dynamic genomic remodeling, with nearly two-thirds of treated patients exhibiting newly acquired alterations. These changes reflect both impaired target engagement via secondary KRAS mutations or copy number gains, and reactivation of downstream signaling through alternative RAS isoforms, non-V600E BRAF mutations, or amplification of RTKs such as EGFR, FGFR2 and MET. In some cases, the disappearance of the KRAS^G12C^ allele further supports clonal replacement by resistant tumor subpopulations^20^. Together, these clinical observations indicate that allele-specific inhibition is vulnerable to both on-target escape and pathway-level rewiring driven by tumor heterogeneity.

Beyond genetic alterations, adaptive signaling reactivation can arise through upstream or parallel pathway engagement. Activation of NRAS or HRAS can restore MAPK signaling despite KRAS inhibition, and such bypass signaling may be partially mitigated by vertical inhibition strategies combining KRAS inhibitors with EGFR or MEK inhibitors^21^. In addition, phenotypic plasticity contributes to variable drug sensitivity. In KRAS^G12C^ inhibitor-treated models, mesenchymal-like states have been associated with reduced responsiveness, in part through sustained PI3K signaling driven by IGFR–IRS1-mediated maintenance of AKT phosphorylation^22^. These findings emphasize that resistance can reflect not only discrete genomic lesions but also state-dependent rewiring of signaling dependencies.

Co-occurring genomic alterations further modulate therapeutic response and clinical outcomes. In NSCLC treated with KRAS^G12C^ inhibitor monotherapy, mutations in *KEAP1*, *SMARCA4* and *CDKN2A* have been associated with poorer outcomes^23^. KEAP1 loss, for example, stabilizes NRF2 and enhances antioxidant defense programs linked to therapeutic resistance; consistent with this, KEAP1 disruption can confer resistance in preclinical models treated with KRAS^G12C^ inhibitors^24^. Collectively, these observations indicate that suppressing KRAS catalytic activity alone is often insufficient to achieve durable pathway control, and that complementary approaches capable of constraining signaling robustness and adaptive rewiring are required.

**Fig. 1:**
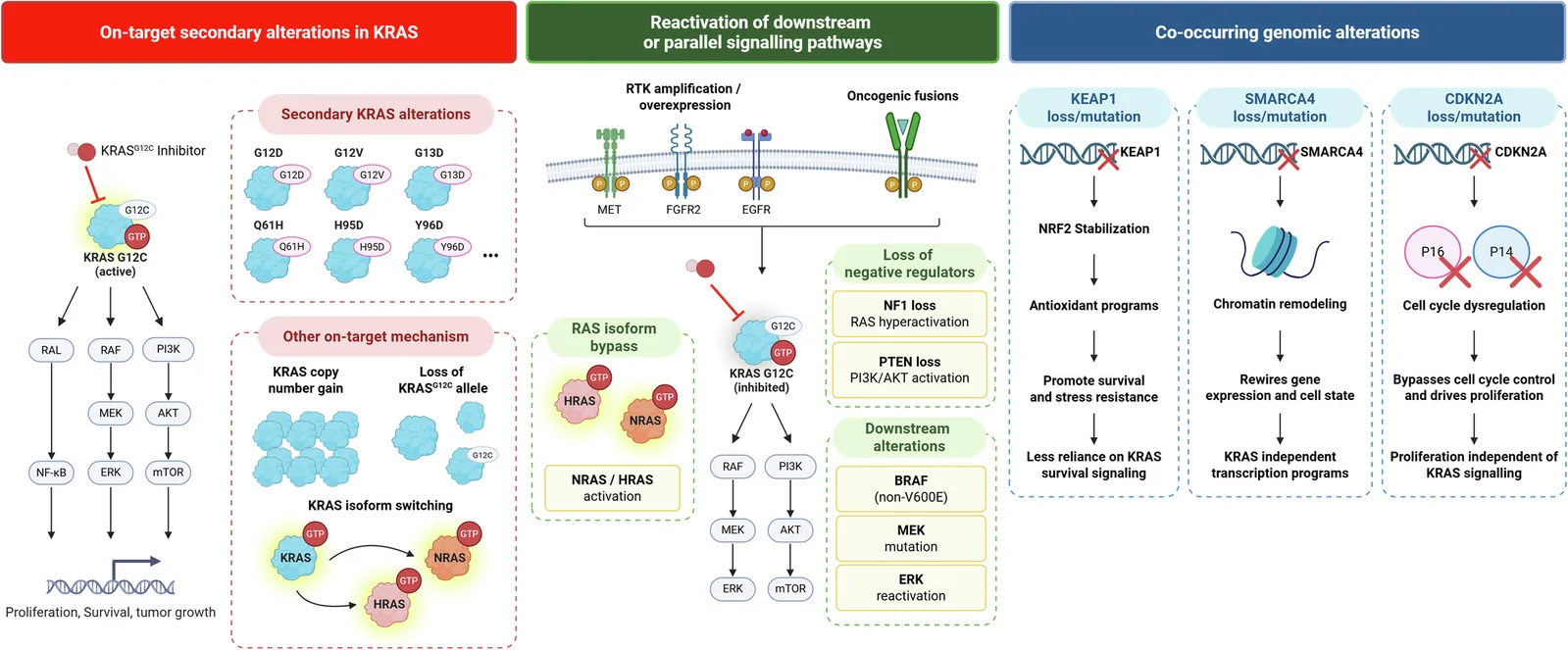
KRAS inhibitor resistance mechanism. KRAS inhibitor resistance can arise through on-target secondary alterations in KRAS, reactivation of downstream or parallel signaling pathways, and co-occurring genomic alterations that reshape pathway dependencies. On-target mechanisms include secondary KRAS mutations, KRAS copy number gain, loss of the KRAS^G12C^ allele, and activation of alternative RAS isoforms. Signaling reactivation may occur through RTK amplification or overexpression, oncogenic fusions, loss of NF1 or PTEN, or downstream alterations in BRAF, MEK and ERK. In parallel, co-occurring alterations such as *KEAP1*, *SMARCA4* and *CDKN2A* loss or mutation can promote stress adaptation, transcriptional plasticity and cell-cycle dysregulation, thereby reducing reliance on KRAS-driven signaling. Together, these mechanisms explain why direct KRAS inhibition alone often fails to achieve durable pathway control.

Multiple combination strategies are therefore under active investigation, including RTK inhibitors upstream of KRAS, inhibitors targeting downstream RAS effector pathways, immune checkpoint blockade and conventional chemotherapies^3^. Several regimens have demonstrated clinical benefit. For example, in KRAS^G12C^-mutant colorectal cancer, combinations targeting both KRAS and EGFR—such as sotorasib plus panitumumab or adagrasib plus cetuximab—have been approved as second-line treatment options^13^. However, similar combinations have not yet achieved broad approval in other tumor types, including lung cancer. Moreover, multidrug resistance can still emerge under combination pressure, highlighting fundamental limitations of approaches that rely exclusively on suppressing KRAS-driven signaling nodes (Fig. 1).

### Rationale for protein-level regulation

Because oncogenic signaling depends not only on enzymatic activity but also on protein abundance, localization and proteostasis, strategies that regulate KRAS pathway components at the protein level represent a mechanistically distinct complement to direct KRAS inhibition. Protein homeostasis is tightly controlled by cellular quality control systems that maintain appropriate protein levels and conformational integrity. Among these, the ubiquitin–proteasome system (UPS) plays a central role in regulated protein turnover and degradation^25^.

### UPS and therapeutic targeting

In the UPS, substrate proteins are modified with ubiquitin chains that encode signals for proteasomal recognition and degradation. Ubiquitination is mediated by an enzymatic cascade involving E1 activating enzymes, E2 conjugating enzymes and E3 ligases that confer substrate specificity. Conversely, deubiquitinases (DUBs) remove ubiquitin chains, counteracting degradation and modulating stability, localization and signaling competence of diverse substrates. Through this reversible regulation, UPS functions not only as a protein disposal pathway but also as a dynamic controller of signaling network architecture^25^.

Therapeutically, modulating ubiquitin signaling has emerged as a promising approach to constrain oncogenic programs. Inhibition of selected DUBs can promote ubiquitination and turnover of oncogenic or prosurvival proteins, thereby sensitizing tumor cells to cell death. In parallel, targeted protein degradation technologies such as proteolysis-targeting chimeras (PROTACs) have demonstrated that forced ubiquitination of disease-associated proteins via E3 recruitment can be pharmacologically exploited. Together, these advances support the concept that UPS-directed strategies—particularly DUB inhibition—can serve as complementary interventions to KRAS inhibitors by regulating protein stability and network robustness through mechanisms distinct from direct catalytic blockade^25^.

## DUBs and E3 ligases regulating KRAS directly

Unlike conventional KRAS inhibitors that primarily suppress KRAS activity, ubiquitin-dependent regulation can directly control KRAS protein abundance, stability, and subcellular localization. Recent studies have identified several DUBs and E3 ligases that govern KRAS turnover, thereby influencing the total signaling capacity of oncogenic KRAS independently of its catalytic state. Beyond turnover, several of these enzymes also influence KRAS membrane localization and signaling competence. In this section, we focus on DUBs and E3 ligases that act directly on the KRAS protein itself^26,27^.

### DUBs that regulate KRAS protein stability

USP7 is one of the best-characterized DUBs directly regulating KRAS. In NSCLC cells, USP7 interacts with KRAS through its TRAF domain and removes K48-linked polyubiquitin chains at lysine 147, thereby enhancing KRAS protein stability independently of its nucleotide-binding state. USP7 expression correlates positively with KRAS levels in patient samples, and pharmacological inhibition of USP7 reduces KRAS stability, attenuates ERK and AKT signaling, and suppresses tumor growth, particularly in models resistant to KRAS^G12C^ inhibitors^28^.

USP2 has also been reported to stabilize KRAS in multiple myeloma cells by removing K48-linked polyubiquitin chains and preventing proteasomal degradation. Pharmacological inhibition or genetic depletion of USP2 promotes KRAS degradation, suppresses tumor cell proliferation, and induces apoptosis both in vitro and in xenograft models^29^.

JOSD2 similarly functions as a KRAS-stabilizing DUB across multiple oncogenic KRAS mutants. JOSD2 directly binds KRAS and removes K48-linked ubiquitin chains in a catalytic-activity-dependent manner. Notably, oncogenic KRAS suppresses the E3 ligase activity of CHIP toward JOSD2, forming a positive feedback loop that enhances KRAS stability and signaling. Inhibition of JOSD2 selectively impaired the growth of KRAS-mutant colorectal cancer cells and patient-derived xenografts^30^.

In addition to these DUBs that directly bind and deubiquitinate KRAS, other DUBs regulate KRAS stability through scaffold-mediated mechanisms. USP9X directly deubiquitinates KRAS in a process mediated by the scaffold protein NDRG3, which recruits USP9X to KRAS. Loss of either USP9X or NDRG3 enhances KRAS ubiquitination, reduces KRAS protein levels and suppresses tumor growth^31^.

Collectively, these studies indicate that multiple DUBs converge on regulating KRAS protein stability, and that pharmacological disruption of these interactions can reduce KRAS abundance and attenuate downstream signaling, supporting the concept that modulation of protein turnover represents a complementary approach to catalytic inhibition.

### DUBs regulating KRAS localization or signaling competence

In addition to regulating protein stability, some DUBs influence KRAS signaling by altering localization or signaling competence.

USP25 regulates oncogenic KRAS through a noncanonical mechanism largely independent of its DUB activity. Rather than altering KRAS stability, USP25 promotes plasma membrane localization of KRAS4B mutants by antagonizing RNF31-mediated linear ubiquitination, thereby preserving oncogenic KRAS signaling^32^.

USP18 has also been implicated in regulating KRAS stability. Loss of USP18 reduces KRAS protein levels without affecting mRNA expression, shortens KRAS half-life, and disrupts plasma membrane localization, thereby impairing downstream signaling. In genetically engineered mouse models, USP18 deficiency suppressed KRAS-driven lung tumorigenesis^33^.

These findings suggest that ubiquitin-dependent regulation of KRAS extends beyond protein degradation and includes control of subcellular localization and signaling competence, providing additional regulatory nodes that may be therapeutically exploitable.

### E3 ligases regulating KRAS turnover

Several E3 ubiquitin ligases have been reported to promote KRAS ubiquitination and degradation. The CUL3–LZTR1 E3 ligase complex is one of the best-characterized examples and facilitates proteasomal degradation of KRAS; therefore, loss of LZTR1 leads to KRAS accumulation and enhanced MAPK signaling^34^. Other ligases, such as ANAPC2^35^, β-TrCP1^35,36^ and NEDD4-1^37^, have also been implicated in regulating KRAS turnover in specific signaling or phosphorylation contexts.

In addition to endogenous ligases, engineered strategies have demonstrated that targeted ubiquitination of KRAS is feasible. Synthetic recruitment of E3 ligases such as Von Hippel-Lindau (VHL) using PROTACs^38^ or expression of chimeric E3 ligase^39^ has shown that forced degradation of mutant KRAS can be achieved experimentally^38^, providing further proof of principle that modulation of KRAS protein stability is a potential therapeutic approach.

Taken together, these observations indicate that both endogenous ubiquitin-regulatory enzymes and engineered degradation approaches can directly influence KRAS protein levels. This layer of regulation provides a mechanistically distinct intervention point that may complement direct inhibition strategies by reducing signaling capacity through changes in protein abundance.

## DUBs and E3 ligases regulating KRAS signaling pathways

In addition to directly regulating the KRAS protein, ubiquitination and deubiquitination also modulate multiple signaling pathways, both upstream and downstream of KRAS. These regulatory mechanisms affect signaling amplitude, duration and pathway reactivation, all of which contribute to tumor progression and therapeutic resistance. While several E3 ligases have been reported to regulate components of KRAS signaling (Table 1), emerging evidence indicates that DUBs are important regulators of pathway activity and potential therapeutic targets (Table 2).

**Table 1:** E3 ligases regulating KRAS signaling.

E3 ligase	Type	Target protein	Ub site/Ub chain	Effects	Cell type	Reference
BIRC6	HECT-like	KRAS4A	Lys128, Lys147	Mono/di-ubiquitination of KRAS4A, affecting GTP loading and growth stimulation of KRAS4A	HEK293, HeLa, U2OS	^87^
HACE1	HECT type	YAP1	ND	Induction of YAP1 downregulation, thereby increasing the activity of Hippo pathway	CRC	^68^
NEDD4-1	HECT type	pan-RAS, PTEN	Lys5 (KRAS)	Degradation; negative feedback between Nedd4-1 and Ras signaling	HEK293T, CRC	^37^
SMURF2	HECT type	KRAS	ND	Positive regulation of KRAS stability; inhibits β-TrCP1-mediated KRAS degradation	NSCLC	^36^
SMURF2	HECT type	EGFR	Lys721, Lys846, Lys1037, Lys1164; K63-linked	Stabilization of EGFR mutant by K63-ubiquitination; competition between acetylation-mediated receptor internalization	NSCLC	^46^
ANAPC2	RING type	KRAS	ND	Upregulated by CRC treatment; degradation of KRAS	CRC	^35^
Cbl	RING type	EGFR	ND	EGFR ubiquitination, downregulation and lysosomal degradation	HEK293, COS-7	^45^
Cbl	RING type	FRS2α	ND	Degradation of FRS2α, downstream adaptor protein for FGFR signaling	HeLa	^88^
Cbl-b	RING type	SHP2	ND	Ubiquitination of SHP2 upon activation	T cells	^89^
CUL3–LZTR1 complex	RING type	KRAS	ND	Degradation of KRAS; oncogenic KRAS mutants disturbs abrogate their association with LZTR1, affecting turnover	HEK293T	^34^
FBXL6	RING type	KRAS, KRAS G12D	Lys128; K63-linked	Activation of KRAS by K63-ubiquitination at K128	HCC	^90^
FBXW7	RING type	mTOR	ND	Degradation of mTOR	Breast cancer cells	^91^
RABGEF1(Rabex-5)	RING type	HRAS, NRAS	ND	Endosomal localization of HRAS and NRAS	HEK293T, COS-1	^92^
SIAH1	RING type	AKT, YAP	ND	Degradation of AKT, YAP; regulation of MAPK, PI3K–AKT and Hippo pathway	CRC	^67^
TRAF6	RING type	AKT	Lys8, Lys14; K11, K63-linked	K63 ubiquitination of AKT, enhancing membrane localization and activity	HEK293T, COS-1, PC-3, MEF	^93^
TRIM4	RING type	TPL2	Lys415, Lys439	Degradation of TPL2; mutant KRAS recruits RNF185, degrading TRIM21 and TRIM4 subsequently	HEK293T	^58^
WDR76	RING type	pan-RAS	ND	Degradation of RAS proteins	HCC	^94^
β-TrCP1	RING type	KRAS	ND	Degradation of KRAS; degradation by SMURF2–UBCH5 complex	NSCLC	^36^
CHIP	U-box type	RAF1	ND	Recruited by XIAP, interacting with RAF1-HSP90 complex	HeLa, SH-SY5Y, HEK293T	^95^

*ND* not determined, ubiquitin chain linkage specificity has not been experimentally validated.

**Table 2:** DUBs regulating KRAS signaling.

DUB	DUB family	Target	Ubiquitin chain	Related protein in KRAS signaling	Effects	Cancer type	Reference
EIF3H	JAMM	HAX1	ND	ERK1/2	Stabilization of HAX1 by antagonizing βTrCP1-mediated ubiquitination, potentiating phosphorylation of ERK1/2	CRC	^54^
MINDY2	MINDY	ACTN4	K48-linked	AKT/mTOR pathway	Stabilization of ACTN4; activates PI3K/AKT/mTOR signaling pathway	PC	^66^
JOSD2	MJD	KRAS	K48-linked	KRAS	Stabilization of KRAS; KRAS inhibits CHIP-mediated JOSD2 degradation.	CRC	^30^
OTUB1	OTU	pan-RAS	ND	pan-RAS	Regulation of RAS localization	NSCLC, CRC, prostate cancer	^96^
OTUD1	OTU	AKT	ND	AKT	Noncanonical inhibition of membrane localization of AKT	Multiple cancer cells	^59^
OTUD1	OTU	PTEN	K48-linked	AKT	Stabilization of PTEN (negative regulator of AKT)	RCC	^59^
OTUD5	OTU	PTEN	ND	AKT	Stabilization of PTEN (negative regulator of AKT)	NSCLC	^59^
OTUD5	OTU	RNF186	ND	mTORC1	Stabilization of E3 ligase RNF186, leading to the degradation of sestrin2 (inhibitor of mTOR signaling)	Bladder cancer cells	^65^
OTUD5	OTU	β-TrCP1	ND	mTOR	Stabilization of β-TrCP1, leading to the degradation of DEPTOR (inhibitory protein of mTORC1/2)	CRC	^65^
OTUD7B	OTU	EGFR	ND	EGFR	Stabilization of EGFR; cancer proliferation	Breast cancer cells	^41^
OTUD3	OTU	PTEN	K6, K11, K27, K48-linked	AKT/mTOR pathway	Stabilization of PTEN; PTEN/AKT pathway suppression	Breast cancer cells	^60^
UCHL5	UCH		ND	AKT/mTOR pathway	Activates AKT/mTOR signaling	Breast cancer cells	^62^
CYLD	USP	AKT	K63-linked	AKT	Inactivation of AKT by K63 deubiquitination	Multiple cancer cells	^59^
USP1	USP	PHLPP1	ND	AKT	Stabilization of PHLPP1 (AKT phosphatase)	NSCLC	^59^
USP2	USP	EGFR	ND	EGFR	Deubiquitination of EGFR at early endosomes; inhibition of endocytosis-mediated EGFR degradation	NSCLC	^42^
USP2	USP	KRAS	K48-linked	KRAS	Stabilization of KRAS	Multiple myeloma	^29^
USP7	USP	AKT	ND	AKT	Stabilization of AKT; recruited by lncRNA USP27X-AS1	HCC	^59^
USP7	USP	PTEN	ND	AKT/mTOR pathway	Regulation of PTEN stability and localization	MEF	^60^
USP7	USP	KRAS	K48-linked (Lys147)	KRAS	Stabilization of KRAS	NSCLC	^28^
USP7	USP	RAF1	M1, K6, K11, K27, K33, K48	RAF1	Stabilization of RAF1	NSCLC	^56^
USP8 (UBPY)	USP	EGFR	ND	EGFR	Regulation of early endosomal sorting of EGFR between degradation and recycling	HeLa	^40^
USP9X	USP	Eps15	ND	EGFR	Inhibition of EGFR internalization by monoubiquitinated endocytosis protein Eps15	HeLa	^44^
USP9X	USP	KRAS	ND	KRAS	Scaffolding protein NDRG3 dependent KRAS deubiquitination	PDAC	^31^
USP10	USP	PTEN	K63-linked	AKT/mTOR pathway	Activation of PTEN by preventing TRIM25-mediated ubiquitination	NSCLC	^60^
USP11	USP	PTEN	ND	AKT/mTOR pathway	Regulatory feedforward loop with PTEN	Breast cancer cells, CRC	^60^
USP13	USP	PTEN	ND	AKT/mTOR pathway	Stabilization of PTEN; PTEN/AKT pathway suppression	Breast cancer cells, OSCC	^60^
USP17	USP	pan-RAS	ND	pan-RAS	Regulation of RAS localization	HeLa, breast cancer cells	^96^
USP18	USP	KRAS	ND	KRAS	Localization of KRAS to plasma membrane and stabilization	NSCLC	^33^
USP21	USP	MEK2	K48-linked	MEK2	Stabilization of MEK2	HCC	^52^
USP22	USP	EGFR	ND	EGFR	Promotion of EGFR recycling after EGF stimulation	lung ADC	^43^
USP25	USP	KRAS	ND	KRAS mutants	Inhibition of RNF31–KRAS mutant interaction indepently of its DUB activity; enhances plasma membrane localization of KRAS mutant and KRAS downstream signaling pathway	NSCLC	^32^
USP28	USP	BRAF	ND	BRAF	Stabilization of E3 ligase FBW7; enhances the degradation of FBW7 substrate BRAF	Melanoma cells	^57^
USP32	USP	BAG3	K48-linked	RAF/MEK/ERK pathway	Stabilization of BAG3; activation of RAF–MEK–ERK signaling pathway	NSCLC	^55^
USP33	USP	RALB	ND	RALB	Deubiquitination of RALB; modulate complex formation between RALB–SEC5 or RALB–EXO84	HEK293T, HeLa	^69^
USP39	USP	Rictor mRNA	ND	mTORC2	Enhancement of selective splicing and maturation of mTOR2 component Rictor mRNA	ESCC	^63^
USP44	USP	PTEN	ND	AKT/mTOR pathway	Stabilization of PTEN; PTEN/AKT pathway suppression	Breast cancer cells, OSCC	^60^
USP47	USP	MAPK	ND	MAPK	Stabilization of MAPK		^53^
USP49	USP	FKBP51	ND	AKT	Stabilization of FKBP51, a scaffolding protein of AKT and PHLPP	PDAC	^61^

*ND* not determined, ubiquitin chain linkage specificity has not been experimentally validated.

### Regulation of RTK–KRAS signaling

Upstream RTKs and GEFs are key determinants of KRAS activation, and several DUBs regulate the stability, trafficking or activity of these upstream regulators.

USP8 (UBPY) is a well-characterized DUB that stabilizes EGFR by preventing lysosomal degradation and promoting receptor recycling, thereby prolonging downstream signaling^40^. Similarly, OTUD7B (Cezanne-1) inhibits EGFR ubiquitination and maintains receptor signaling, and its overexpression correlates with poor prognosis in multiple cancers^41^. USP2^42^ and USP22^43^ have also been reported to stabilize EGFR and sustain downstream signaling pathways, including AKT and STAT3, contributing to tumor growth and therapy resistance. In addition, USP9X indirectly regulates EGFR trafficking by deubiquitinating the endocytic adaptor EPS15, thereby affecting receptor internalization and downstream KRAS signaling^44^. E3 ligases also regulate upstream receptors. For example, c-Cbl negatively regulates EGFR by promoting receptor ubiquitination and lysosomal degradation, thereby limiting KRAS activation^45^. However, the functional outcomes of E3 ligases in EGFR regulation are not uniformly suppressive and can vary depending on context and mechanism. By contrast, the E3 ligase SMURF2 promotes ubiquitination and stabilizes EGFR by inhibiting lysine-acetylation-mediated internalization of the EGFR^46^.

Beyond receptor regulation, the upstream activators of KRAS are themselves subject to ubiquitin-dependent control. Proteins such as SOS1^47^, GRB2^48^ and SHP2^49^ have been reported to undergo ubiquitination and, in certain contexts, proteasomal degradation, highlighting the importance of ubiquitin-dependent regulation in controlling RAS pathway activation. Pharmacological inhibition of these upstream components has demonstrated efficacy in KRAS-mutant cancers^50,51^, underscoring their functional relevance. Although the specific DUBs that oppose this degradation have not yet been clearly identified, the existence of ubiquitin-dependent control suggests that DUB-mediated regulation of these activators may remain to be discovered, representing an additional therapeutic layer worth exploring.

Taken together, these findings indicate that ubiquitin-dependent regulation of upstream signaling components can sustain or restrain KRAS activation. Stabilization of RTKs or GEFs by DUBs may maintain signaling even in the presence of KRAS inhibitors, highlighting these enzymes as potential targets to limit adaptive pathway reactivation.

### Regulation of the RAF–MEK–ERK pathway

The RAF–MEK–ERK cascade is a major effector pathway downstream of KRAS and represents a frequent route of adaptive resistance^2^. Several DUBs directly regulate components of this pathway. USP21 stabilizes MEK2 by removing K48-linked ubiquitin chains, thereby enhancing ERK activation and promoting tumor growth^52^. USP47 has been reported to stabilize MAPK pathway components by counteracting ubiquitin-mediated degradation, sustaining ERK signaling^53^.

Other DUBs influence MAPK signaling indirectly through regulation of scaffold or regulatory proteins. EIF3H stabilizes HAX1, thereby enhancing RAF–MEK–ERK complex assembly and amplifying ERK activation^54^. Similarly, USP32 indirectly activates the pathway by stabilizing BAG3, which promotes RAF–MEK–ERK complex formation and enhances ERK phosphorylation in lung cancer models^55^. DUBs also regulate RAF kinases themselves. USP7 has been reported to deubiquitinate RAF1 in certain contexts^56^, while USP28^57^ has been implicated in stabilizing BRAF and RAF complex components, supporting sustained MAPK pathway activity.

Several E3 ligases counterbalance these effects; for example, TRIM4 targets TPL2 (MAP3K8), a MEK–ERK activating kinase functioning downstream or in parallel to KRAS, for degradation, thereby limiting ERK activation. Loss of TRIM4 results in enhanced MAPK signaling^58^. Similarly, ubiquitin-mediated degradation of BRAF by E3 ligases such as FBXW7 and RNF149 has been shown to restrain MAPK signaling, further highlighting the importance of UPS-dependent regulation throughout the RAF–MEK–ERK cascade.

Together, these studies show that ubiquitin-mediated regulation acts at multiple points in the MAPK cascade, affecting both signal strength and duration. This layered control adds more regulatory points that could be targeted to prevent pathway reactivation in KRAS-driven tumors.

### Regulation of the PI3K–AKT–mTOR pathway

The PI3K–AKT–mTOR pathway is another major effector of KRAS signaling and plays a central role in cell survival, metabolism and therapeutic resistance. Multiple DUBs regulate this pathway through distinct mechanisms. USP1 suppresses AKT activation by removing K63-linked ubiquitin chains required for AKT phosphorylation, whereas USP7 stabilizes AKT and maintains pathway activation, illustrating that DUBs can exert both positive and negative regulatory effects^59^. Several DUBs regulate PTEN, a key negative regulator of PI3K signaling. USP7, USP10, USP13 and USP44 modulate PTEN’s stability, localization, or activity, thereby regulating PI3K–AKT signaling^60^. Loss or dysregulation of these DUBs can therefore lead to hyperactivation of the pathway.

Additional mechanisms have also been identified. USP49 suppresses AKT signaling by stabilizing FKBP51, which promotes AKT dephosphorylation^61^, whereas UCHL5 enhances AKT/mTOR signaling by stabilizing pathway components and promoting c-Myc expression^62^. USP39, although lacking canonical DUB activity, indirectly activates mTORC2 signaling and has been linked to chemoresistance^63^. Other DUBs, including OTUD5 and MINDY2, activate mTOR signaling by stabilizing regulators such as RNF186^64^, β-TrCP1^65^ or ACTN4^66^, further illustrating the diversity of ubiquitin-mediated regulation within this pathway.

E3 ligases also play a role in regulating pathways. SIAH1^67^ and HACE1^68^ have been reported to suppress AKT or YAP signaling through ubiquitin-mediated degradation of pathway components, acting as tumor suppressors in certain KRAS-driven cancers.

Overall, these findings indicate that ubiquitin-mediated regulation of the PI3K–AKT–mTOR pathway plays a critical role in determining survival signaling and therapeutic response, and that perturbation of DUB activity may alter pathway dependencies in KRAS-driven tumors.

### Regulation of other KRAS effector pathways

Beyond MAPK and PI3K signaling, DUBs also regulate additional KRAS effector pathways. USP33 regulates RALB ubiquitination and functions as a molecular switch that controls autophagy and innate immune signaling, depending on the cellular context. This demonstrates the broader role of ubiquitin signaling in shaping KRAS-driven cellular phenotypes^69^.

Other pathways, including Wnt signaling, are also influenced by ubiquitin ligases and DUBs that indirectly regulate tumor progression. Increasing evidence indicates that KRAS signaling functionally intersects with the Wnt pathways through coordinated regulation of cell proliferation and metabolic adaptation. In KRAS-driven cancers, aberrant activation of Wnt/β-catenin signaling has been associated with enhanced tumor progression and resistance to targeted therapies^70^. Therefore, ubiquitin-mediated regulators of these pathways may indirectly influence KRAS-dependent tumor behavior and therapeutic response. For example, RNF43 and ZNRF3 suppress Wnt signaling by promoting the degradation of Frizzled receptors^71^, whereas XIAP enhances β-catenin signaling by monoubiquitinating transcriptional regulators^72^. Although these mechanisms are less directly linked to KRAS activation, they may still contribute to tumor progression and therapeutic resistance in KRAS-driven cancers.

**Fig. 2:**
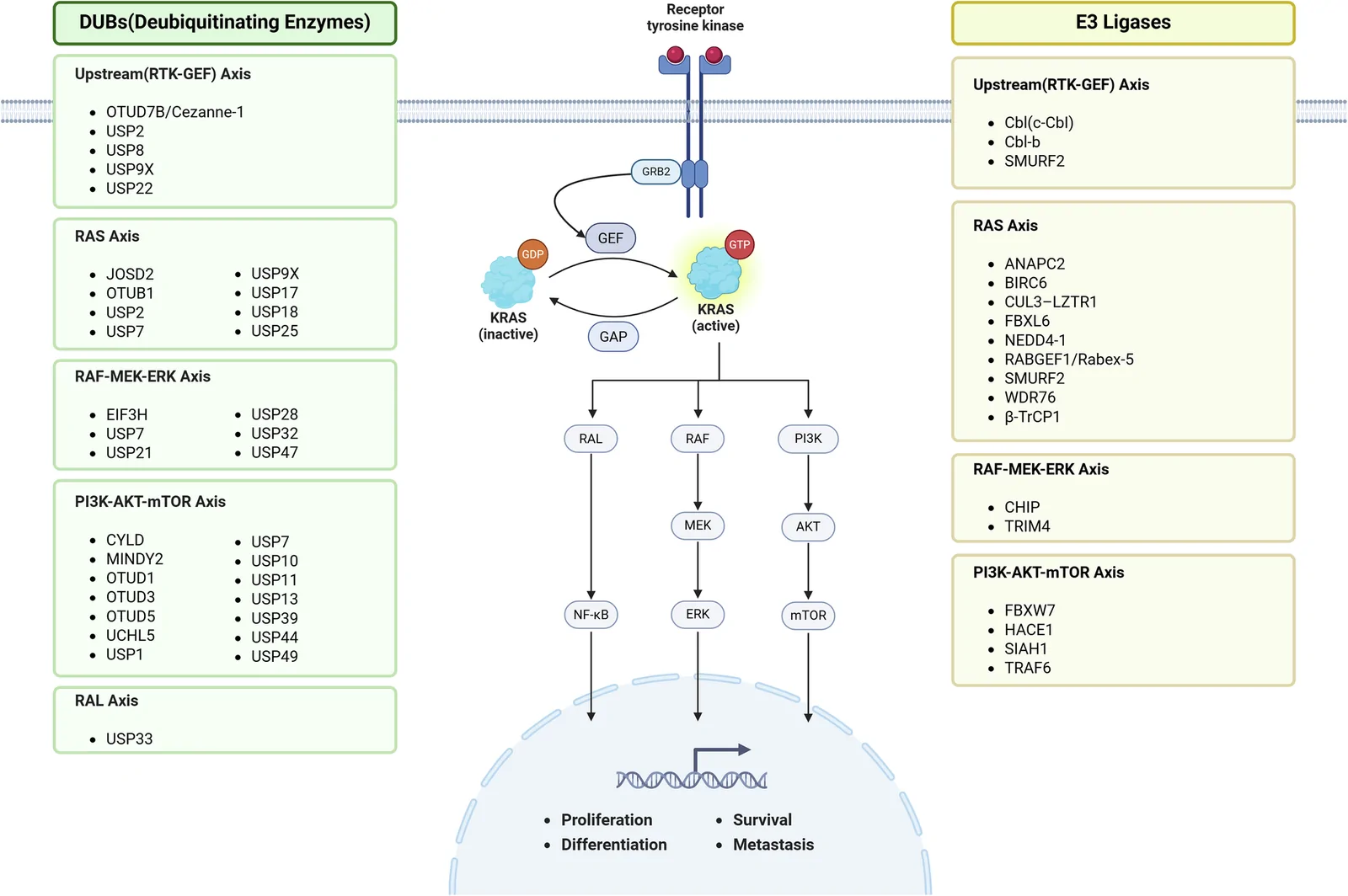
DUB/E3 ligase regulation of the KRAS signaling network. DUBs and E3 ligases regulate multiple nodes of the KRAS signaling network, from upstream RTK–GEF activation to KRAS itself and downstream RAL, RAF–MEK–ERK and PI3K–AKT–mTOR pathways. DUBs generally counteract ubiquitination by removing ubiquitin chains from substrate proteins, thereby modulating protein stability, localization or signaling competence. By contrast, E3 ligases confer substrate-specific ubiquitination and can promote protein turnover or alter signaling activity depending on the substrate and cellular context. Through these opposing but coordinated activities, ubiquitin-regulatory enzymes influence KRAS pathway amplitude, signaling duration and adaptive pathway reactivation in KRAS-driven cancers.

Collectively, these studies demonstrate that ubiquitin-mediated regulation influences KRAS signaling at multiple levels, including upstream activation, downstream effector pathways, and parallel signaling networks. In particular, DUBs regulate KRAS-driven signaling through diverse mechanisms, including modulation of protein stability, trafficking, localization and protein complex assembly. While these functions collectively contribute to pathway adaptation and therapeutic resistance, regulation of protein stability is particularly attractive from a therapeutic perspective because pharmacological DUB inhibition can directly alter the abundance of oncogenic proteins and signaling regulators. Therefore, although DUBs exert both canonical and noncanonical functions, this Review places particular emphasis on DUBs as modulators of protein turnover and their potential exploitation for therapeutic intervention (Fig. 2).

## Therapeutic implications of DUB inhibitors and targeted degradation strategies

### Why DUB targeting is mechanistically distinct

Proteins involved in cell survival, proliferation and signaling must be maintained at appropriate levels and in proper conformational states to ensure normal cellular function. Disruption of this balance leads to the accumulation of misfolded or excess proteins, cellular stress and disease development. UPS is a major pathway responsible for regulated protein degradation and protein quality control.

Unlike conventional inhibitors that suppress catalytic activity, targeting DUBs influences protein stability and turnover. Because this strategy regulates the abundance of the target protein rather than its enzymatic activity, it can reduce signaling robustness and limit compensatory responses, such as feedback activation or increased protein expression.

Importantly, strategies that modulate protein stability are not restricted to mutation-specific binding pockets^27,73^. Consequently, targeting protein turnover may provide a means to address the heterogeneity of KRAS mutations and signaling contexts that can limit the effectiveness of allele-specific inhibitors.

In addition, DUB-mediated regulation frequently occurs at the level of signaling networks^27^. Modulating DUB activity can influence not only KRAS itself but also upstream regulators and downstream effectors, enabling broader control of pathway output rather than inhibiting a single catalytic step.

Importantly, the therapeutic value of DUB inhibition extends beyond protein turnover. As noted above, several DUBs regulate KRAS-pathway components through nondegradative or noncanonical mechanisms—for example, controlling KRAS membrane localization and signaling competence (USP25 and USP18) or acting largely independently of their catalytic activity. Inhibiting these DUBs can disrupt oncogenic signaling through routes that abundance-lowering strategies alone would not capture, providing distinct and complementary points of intervention that broaden the mechanistic scope of DUB targeting.

### Potential advantages of targeting DUB in KRAS inhibitor resistance

Resistance to targeted therapies frequently arises through secondary mutations, pathway reactivation or compensatory transcriptional upregulation of oncogenic proteins. Unlike catalytic blockade, DUB inhibition lowers the steady-state abundance of the target protein rather than competing for a binding pocket, offering several advantages in the resistance setting.

First, because it acts on protein level rather than on a mutation-defined active site, DUB inhibition is expected to remain effective against secondary KRAS mutations and heterogeneous mutational backgrounds that erode allele-specific inhibitors. Second, because many resistance mechanisms restore signaling by increasing protein abundance—via feedback upregulation of RTKs, accumulation of wild-type RAS isoforms, or effector stabilization—degrading these proteins directly counteracts the adaptive dosage increase that drives rebound signaling. Third, because individual DUBs often act at multiple network nodes, their inhibition can simultaneously dampen upstream activators and downstream effectors, constraining the redundancy that single-node inhibitors leave intact. Together, these properties make DUB inhibition a complementary modality that raises the threshold for adaptive escape, particularly when combined vertically with a direct KRAS inhibitor.

Consistent with this rationale, modulation of ubiquitin signaling can impair survival pathways and promote cancer cell death. For example, USP13 depletion destabilizes the pro-survival protein cIAP2 and sensitizes colorectal cancer cells to necroptosis^74^, highlighting the therapeutic potential of DUB inhibition. In addition, ubiquitin-dependent regulation has been implicated in antitumor immunity and immune checkpoint signaling, including modulation of PD-1/PD-L1 pathways, although these effects are highly context dependent^75^.

In some experimental models, DUB inhibitors have demonstrated activity in cancer cells resistant to proteasome inhibitors. This suggests that selectively modulating ubiquitination dynamics can produce distinct biological effects compared with the global inhibition of proteasomal degradation^76^. Notably, pharmacological inhibition of specific DUBs, such as USP7^28^ and USP47^77^, has been shown to suppress oncogenic signaling, either by destabilizing KRAS or by reducing downstream effectors such as c-Myc, and enhance sensitivity to KRAS-targeted therapies, including improved responses to KRAS^G12C^ inhibitors. Together, these observations indicate that targeting DUBs may provide a complementary strategy to address adaptive resistance mechanisms in KRAS-driven cancers (Table 3).

**Table 3:** DUB inhibitors targeting KRAS-associated DUBs.

DUB target	Compound/structure	Biological effects	Cell type	Ref
CYLD	Subquinocin	Enhances activation of NF-κB signaling	HEK293T, HeLa	^97^
JOSD2	HY041004, HY04071	Inhibition of JOSD2-mediated liver kinase B1 (LKB1) deubiquitination	NSCLC	^98^
OTUB1, USP8	Compound 61	Proliferation inhibition	NSCLC	^99^
OTUD3	OTUDin3	Antiproliferation and proapoptotic effects	NSCLC, in vivo	^100^
OTUD7B	Compound 7Bi, compound 29	Cancer cell proliferation inhibition	NSCLC	^101^
USP1	ML323	Accumulation of ubiquitinated PCNA, resulting in DNA damage, S-phase arrest and apoptosis	Breast cancer cell	^102^
USP2	ML364	Increase of cyclin D1 degradation, cell cycle arrest; decrease of DNA repair	HCT116	^103^
USP8	Isoginkgetin	USP8 downregulation and GPX4 instabilization, inducing ferroptosis	Ovarian cancer cells	^104^
USP9X	FT709	Decreased YAP protein expression without affecting mRNA levels; impaired cell motility	Melanoma	^105^
USP10	Wu-5	Inhibition of FLT3 and AMPK pathway	AML	^106^
USP10, USP13	Spautin-1	Induction of G2/M cell cycle arrest and apoptosis via upregulation of reactive oxygen species-mediated DNA damage	Malignant melanoma	^107^
USP11	Mitoxantrone	Instabilization of DNA damage repair protein, BRCA2	PDA	^108^
USP14, UCHL5	b-AP15, VLX1570	Induction of cell cycle arrest and apoptosis	RCC	^109^
USP18	ZHAWOC8655	USP18/ISG15 interaction interfering	HEK293	^110^
USP21	BAY-805	Strong NF-κB activation	HEK293T, Caco-2	^111^
USP22	Usp22i-S02	Downregulation of FoxP3; enhances antitumor immunity	In vivo	^112^
USP25, USP28	AZ1	Induction of DNA damage and mitochondrial apoptosis	NSCLC	^113^
USP32	Huib32	Enhancing substrate ubiquitination, altering endosomal morphology and mimicking USP32 depletion	HEK293T, melanoma	^114^
USP47	K-552	c-Myc destabilization in KRAS G12C NSCLC; increases sotorasib sensitivity	NSCLC	^77^

### Interplay between DUB inhibition and targeted protein degradation

Targeted protein degradation technologies such as PROTACs provide an alternative approach to regulating protein levels. PROTACs are bifunctional molecules that link a target-binding moiety to an E3 ligase-recruiting ligand via a chemical ligand. By recruiting an E3 ligase to a protein of interest, PROTACs induce ubiquitination and proteasomal degradation, enabling the targeting of proteins previously considered undruggable. Because PROTACs act catalytically and eliminate the target protein rather than merely inhibiting its activity, they may achieve high potency at relatively low concentrations and may reduce the likelihood of resistance developing from mutations that impair inhibitor binding^73^.

In addition, compounds targeting the upstream KRAS signaling proteins SOS1^78^ and SHP2^79^ have been linked to ligands of the E3 ligases VHL or CRBN to promote their selective degradation, thereby indirectly suppressing KRAS activity. PROTACs that selectively degrade KRAS^G12D^ mutant^80^ are also being developed, and their efficacy has been demonstrated across cancer models harboring diverse mutations. In addition, PROTACs targeting downstream components of the KRAS signaling pathway, including BRAF^81^, MEK1/2^82^ and PI3K^83^, are being explored as potential strategies to attenuate the effects of hyperactivated KRAS signaling (Table 4). However, PROTACs also face several limitations^84^. Their relatively large molecular size often results in suboptimal pharmacokinetic properties, including limited cell permeability and challenges in oral bioavailability. In addition, the efficacy of PROTACs depends on the expression and activity of the recruited E3 ligase, and alterations in E3 ligase levels or function may compromise therapeutic efficacy. Off-target degradation and toxicity also remain important concerns.

**Table 4:** Targeted protein degradation strategies relevant to KRAS signaling.

Target protein	PROTAC name	Warhead	E3 ligase ligand	Cell type	Ref
EGFR	MS-154	Gefetinib	CRBN	HCC827, H3255	^115^
EGFR	MS39	Gefetinib	VHL	HCC827, H3255	^115^
BRAF V600E	SJF-0628	Vemurafenib	VHL	SK-MEL-28, SK-MEL-239-C4, OVCAR-8	^81^
SHP2	ZB-S-29	Potent SHP2 inhibitor TNO155 analog	CRBN	B myelomonocytic leukemia cell lines	^115^
SHP2	SHP2-D26	Potent SHP2 inhibitor compound 5	VHL	Esophageal cancer cell, macrophage cell lines	^115^
SHP2	P9	SHP9 allosteric inhibitor	VHL / CRBN	HEK293, SKBR3, U2OS, MCF7, A549	^79^
SOS1	SIAIS562055	SOS1 inhibitor BI-3406 analog	VHL / CRBN	Leukemia cells, pancreatic cancer cells, CRC, NSCLC	^78^
pan-RAS	DARPin K27-SPOP	Target-binding domain	E3 ligase adaptor	AsPC-1	^96^
pan-RAS	RC-U	RAS protein effector domain	E3 ligase domain	PANC-1, MIA PaCa-2, AsPC-1, BxPC-3; PANC-1 xenograft mouse model	^96^
pan-RAS	AdPROM	RAS-binding monobody	VHL	A549	^96^
pan-KRAS	ACBI3	KRAS binder (SII-P)	VHL	GP5d, LS513; GP5d xenograft mouse model	^96^
pan-KRAS	ACBI3	KRAS noncovalent ligand compound 3	VHL	GP2d, GP5d, SW620, NCI-H358, HEK293, MIA PaCa-2, HCC461, A549, GEO, HS766T, HCT116, LS513, RKN, B16F10, mouse embryonic fibroblasts, A375	^38^
KRAS	DARPin K19-VHL	MCB-294	VHL	A549, H358, HCT116, HEK293T, HT1080, MIA PaCa2; H358 xenograft mouse model	^96^
KRAS G12C	XY-4-88	ARS-1620	CRBN	MiaPaCa2, H358	^96^
KRAS G12C	KP-14	Acrylamide	CRBN	H358	^96^
KRAS G12C	LC-2	MRTX849	VHL	H2030, MIA PaCa-2, SW1573, H23, H358	^96^
KRAS G12C	YF135	MRTX849	VHL	NSCLC	^96^
KRAS G12C	III-2	AMG-510	VHL	H358	^96^
KRAS G12D	ASP3082	N/A	N/A	mouse model	^96^
KRAS G12D	CH091138 (6)	KRAS G12D inhibitor	VHL	293T, HeLa, H358, A549, BaF3, PC9	^80^
KRAS G12D	8o	MRTX1133 analog	VHL	SNU-1, HPAF-II, AGS, and PANC; AsPC-1 xenograft mouse model	^96^
PI3K	ZM-PI05	Copanlisib	CRBN	Breast cancer cell lines	^83^
MEK1/2	P6b	Coumarin-based MEK inhibitor	VHL	A375, A549	^82^
AKT	MS21	AKT inhibitor	CRBN	BT549, HCT116, PANC1, MiaPaca2 and SK-MEL-239	^116^

**Fig. 3:**
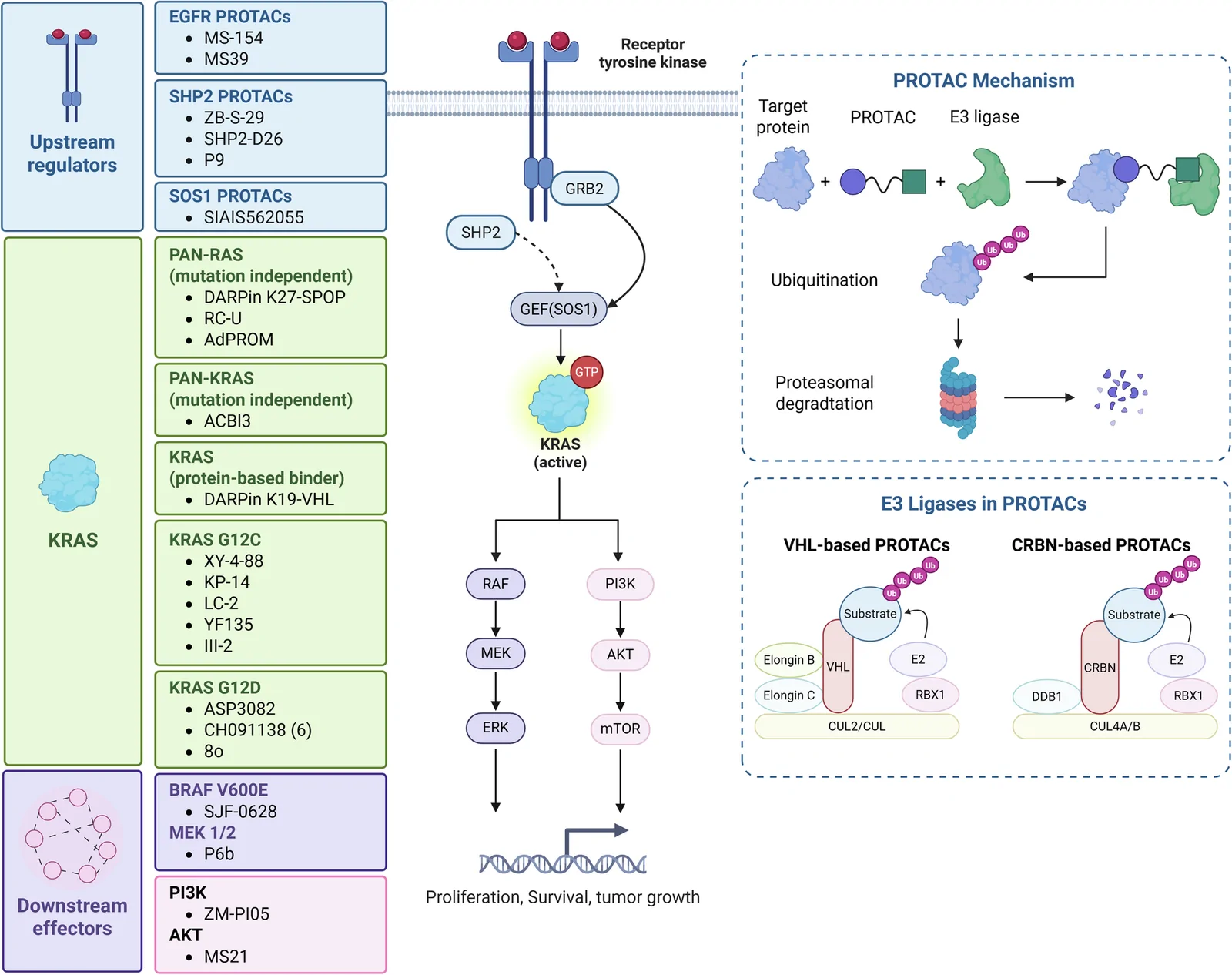
PROTAC/degrader strategies in KRAS signaling. Targeted protein degradation provides an alternative strategy to suppress KRAS-driven signaling by eliminating disease-relevant proteins rather than simply inhibiting their catalytic activity. These molecules induce proximity between a target protein and an E3 ligase, leading to ubiquitination and proteasomal degradation of the target. Degrader strategies have been developed across the KRAS signaling axis, including upstream regulators, KRAS/RAS proteins and downstream effectors, commonly using VHL- or CRBN-based E3 ligase recruitment platforms.

Importantly, these two modalities are mechanistically interconnected, as both converge on the ubiquitin status of the target. Because DUBs oppose the degradative chains a PROTAC installs, a DUB acting on the target or its E3 ligase can blunt degradation and drive resistance—therefore, co-inhibiting that DUB would protect the ubiquitin signal and deepen degradation. Acting on opposite arms of the same equilibrium, DUB inhibition and targeted degradation are thus complementary and potentially synergistic rather than competing (Fig. 3).

## Challenges and future perspectives

Although targeting DUBs represents a promising strategy for regulating KRAS signaling, several challenges must be addressed before this approach can be translated into clinical practice.

### Specificity and substrate complexity

One of the major challenges in developing DUB-targeted therapies is substrate specificity. Many DUBs regulate multiple substrates involved in diverse cellular processes, and conversely, a single signaling protein may be controlled by several DUBs. Although various approaches have been developed to identify DUB substrates in cells, these methods still face several challenges, including limited specificity or sensitivity, technical difficulties and insufficient physiological relevance^85^. This redundancy and network complexity make it difficult to predict the downstream consequences of inhibiting a single DUB. In addition, context-dependent functions of DUBs—including tumor-promoting or tumor-suppressive roles depending on cell type or signaling environment—further complicate therapeutic targeting.

Improved structural understanding of DUB–substrate interactions and the development of highly selective inhibitors will therefore be critical for minimizing off-target effects and achieving therapeutic precision.

### Toxicity and therapeutic window

Another important concern is toxicity. The UPS plays a fundamental role in maintaining protein homeostasis in normal cells, and systemic perturbation of ubiquitin signaling may lead to unintended cellular stress or tissue damage. Compared with proteasome inhibitors, DUB inhibitors may offer greater selectivity; however, the potential for toxicity remains substantial, particularly for broadly expressed or acting DUBs.

Future efforts should focus on developing inhibitors with improved selectivity, context-dependent activity or delivery strategies that specifically target the tumor to broaden the therapeutic window.

### Need for predictive biomarkers

As with many targeted therapies, identifying predictive biomarkers will be essential for the successful clinical development of DUB inhibitors^86^. Expression levels of specific DUBs, dependency of tumor cells on particular ubiquitin-regulated signaling nodes, and co-occurring genomic alterations may all influence therapeutic response.

Systematic profiling of ubiquitin signaling networks in KRAS-driven tumors, combined with functional genomic approaches, may help identify patient subsets most likely to benefit from DUB-targeted therapies.

### Opportunities for combination strategies

Given the complexity of KRAS-driven signaling networks, DUB inhibition is unlikely to replace direct KRAS inhibitors but may provide substantial benefit in combination settings. Targeting DUBs that regulate upstream activators, downstream effectors or parallel pathways may help suppress adaptive signaling and delay resistance. In addition, combining DUB inhibitors with targeted therapies, immunotherapies or protein-degradation strategies, such as PROTACs, may further enhance therapeutic efficacy. Rational combination strategies driven by signaling dependencies and resistance mechanisms will probably represent an important direction for future research.

### Concluding perspective

Accumulating evidence indicates that ubiquitin-mediated regulation plays a critical role in controlling KRAS stability and signaling networks. Although major challenges still remain, ongoing progress in understanding DUB biology, developing inhibitors and discovering biomarkers is expected to expand the therapeutic potential of this approach.

**Fig. 4:**
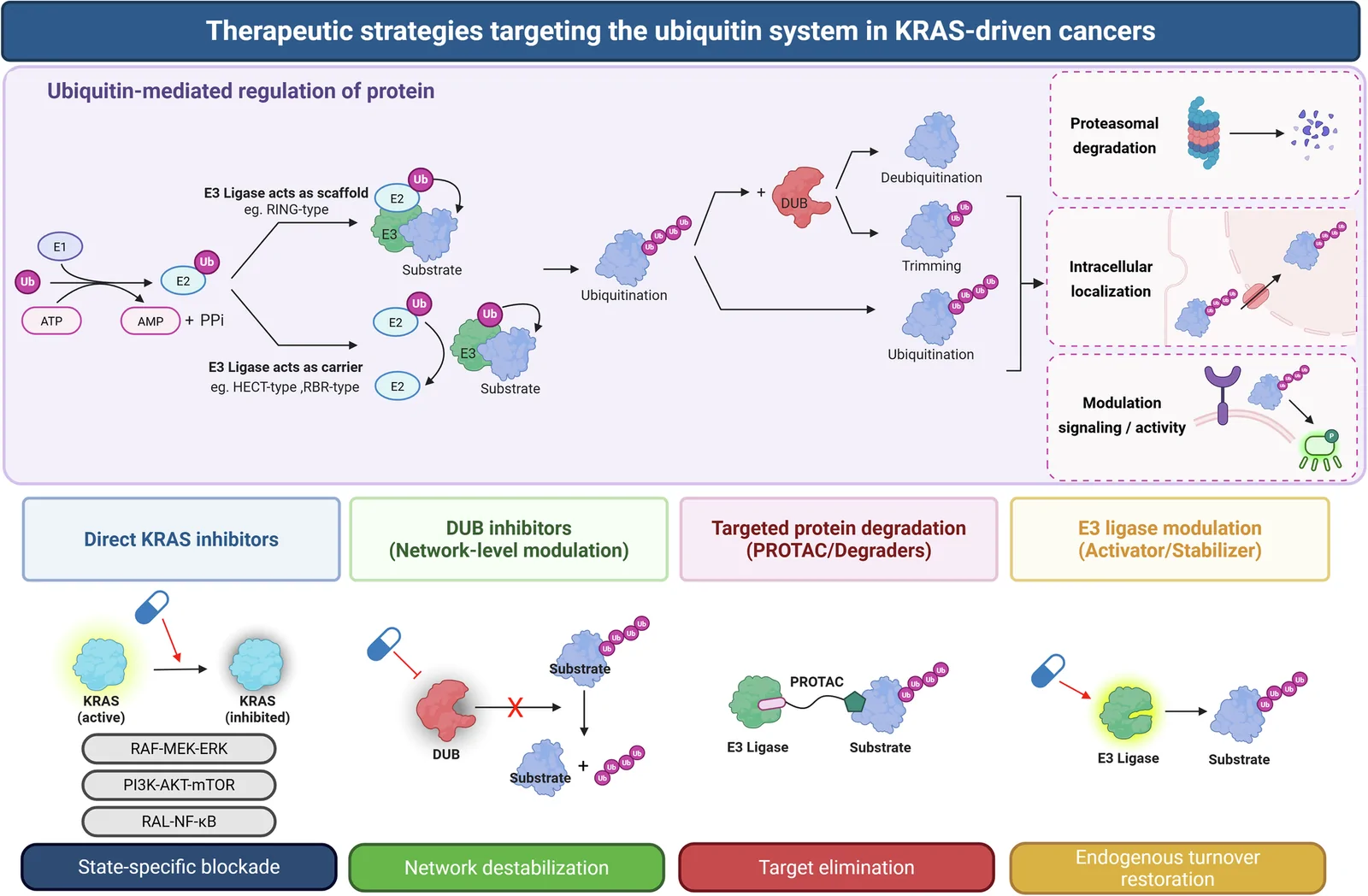
Therapeutic strategies targeting the ubiquitin system in KRAS cancer. The ubiquitin system regulates protein stability, localization and signaling activity through the coordinated actions of E1 activating enzymes, E2 conjugating enzymes, E3 ligases and DUBs. E1–E2–E3 enzyme cascades mediate substrate ubiquitination, whereas DUBs reverse or edit ubiquitin modifications. In contrast to direct KRAS inhibitors, which suppress mutant KRAS activity in a state-specific manner, ubiquitin-directed strategies act at the protein-regulatory level. DUB inhibitors, PROTACs/degraders and E3 ligase modulators may therefore provide complementary approaches to suppress KRAS-driven signaling by modulating protein turnover, target abundance, localization and signaling competence.

Rather than replacing direct KRAS inhibition, DUB targeting is likely to serve as a complementary strategy that enhances the efficacy and durability of KRAS-directed therapies by modulating signaling robustness and adaptive responses (Fig. 4).

## Supplementary information

Supplementary Table 1
