# Author Correction: Rewiring KRAS-driven cancers through the ubiquitin–proteasome system: therapeutic opportunities with a focus on deubiquitinase

**DOI:** 10.1038/s12276-026-01873-2

**Published:** 2026-09-30

**Authors:** Yeonjoo Lee, SuA Hwang, Hyungkyung Shin, Kyeongeun Choi, SeungYeon Seo, HyoKyoung Kim, Ji Su Yang, Yeon Jung Kim, Young-Mee Kim, Eun Joo Song

**Affiliations:** 1 Graduate School of Pharmaceutical Sciences and College of Pharmacy, Ewha Womans University, Seoul, Republic of Korea; 2 Graduate Program in Innovative Biomaterials Convergence, Ewha Womans University, Seoul, Republic of Korea; 3 Department of Biochemistry and Molecular Genetics, University of Illinois Chicago, Chicago, IL USA

**Keywords:** Ubiquitylation, Oncogenes

Correction to: *Experimental & Molecular Medicine* https://doi.org/10.1038/s12276-026-01837-6, published online 07 September 2026

After the online publication of this article, the authors noticed **two errors** in the author information.

First, the equal contribution designation for SuA Hwang was inadvertently omitted. The first two authors, Yeonjoo Lee and SuA Hwang, contributed equally to this work and should have been designated as co-first authors.

These authors contributed equally: Yeonjoo Lee, SuA Hwang

Second, the affiliation of Young-Mee Kim was incorrectly listed as:

“Department of Biochemistry and Molecular Genetics, University of Illinois College of Medicine, Chicago, IL, USA”

The correct affiliation should read:

“Department of Biochemistry and Molecular Genetics, University of Illinois Chicago, Chicago, IL, USA.”

These corrections do not affect the authorship, author order, author contributions, acknowledgements, disclosures, or the scientific content and conclusions of the article.

The authors apologize for any inconvenience caused.

The original article has been corrected.

