# Supplementary material for: Rewiring KRAS-driven cancers through the ubiquitin–proteasome system: therapeutic opportunities with a focus on deubiquitinase
Source: Exp Mol Med. 2026 Sep 7;58(9):2844–58. doi: 10.1038/s12276-026-01837-6 (PMC13633255; doi:10.1038/s12276-026-01837-6)
Supplement: Supplementary file 1 [file 12276_2026_1837_MOESM1_ESM.pdf]

Supplementary Table 1. FDA-approved and clinically investigated small-molecule inhibitors targeting KRAS.

Compound	Sponsor/Company	KRAS mutant	Developmental status	Disease
Adagrasib (Krazati)	Bristol Myers Squibb	G12C	FDA approval	NSCLC, CRC
AN9025	Adlai Nortye Biopharma Co., Ltd.	pan-RAS(ON)	Phase 1	Advanced or metastatic solid tumors
BBO-11818	TheRas, Inc., BridgeBio Oncology Therapeutics	pan-KRAS	Phase 1	Advanced solid tumors
BI-2865	Boehringer Ingelheim	pan-KRAS	Non-clinical	
BLU-924 (SAR449336)	Blueprint Medicines Corporation	pan-KRAS	Phase 1, 2	Advanced PDAC, NSCLC, CRC
D-1553 (Garsorasib)	Guangdong Provincial People's Hospital	G12C selective	Phase 2	NSCLC
	Sun Yat-sen University		Phase 2	Advanced or metastatic NSCLC
	InxMed (Shanghai) Co., Ltd.		Phase 3	Advanced or metastatic NSCLC
GDC-6036 (Divarasib)	Hoffmann-La Roche	G12C	Phase 1	Metastatic CRC
			Phase 3	NSCLC
			Phase 3	Advanced or metastatic NSCLC
IBI351 (Fulzerasib)	Guangdong Association of Clinical Trials	G12C	Phase 2	Advanced or metastatic NSCLC
	Zhejiang University		Phase 2	Advanced CRC
INCB161734	Incyte Corporation	G12D	Phase 1	Advanced or metastatic solid tumors
			Phase 3	Metastatic PDAC
JAB-21822 (Glecirasib)	Peking University Cancer Hospital & Institute	G12C	Phase 1	Metastatic CRC
	Cancer Institute and Hospital, Chinese Academy of Medical Sciences		Phase 1, 2	Advanced or metastatic NSCLC
	Abbisko Therapeutics Co, Ltd		Phase 2	Advanced or metastatic NSCLC
JAB-23E73	Jacobio Pharmaceuticals Co., Ltd.	pan-KRAS	Phase 1, 2	Metastatic PDAC
			Phase 1, 2	Advanced solid tumors
			Phase 1, 2	Advanced solid tumors
JDQ443 (Opnurasib)	Novartis Pharmaceuticals	G12C	Phase 2	NSCLC
LY3962673	Eli Lilly and Company	G12D	Phase 1	Solid tumors
MRTX1133	Mirati Therapeutics Inc.	G12D	Phase 1	Advanced solid tumors
QTX3046	Quanta Therapeutics	G12D	Phase 1	Advanced solid tumors

RMC-5127	Revolution Medicines, Inc.	G12V	Phase 1	Advanced solid tumors
RMC-6236 (Daraxonrasib)	Revolution Medicines, Inc.	pan-RAS(ON)	Phase 3	Metastatic PDAC
			Phase 3	PDAC
RMC-9805 (Zoldonrasib)	Tango Therapeutics, Inc.	G12D	Phase 1, 2	PDAC, NSCLC
	Revolution Medicines, Inc.		Phase 1, 2	Solid tumors
			Phase 3	Metastatic PDAC
Sotorasib (Lumakras)	Amgen	G12C	FDA approval	NSCLC, CRC
TSN1611	Tyligand Pharmaceuticals (Suzhou) Limited	G12D	Phase 1, 2	Advanced solid tumors
VS-7375	Verastem, Inc.	G12D	Phase 2	CRC
			Phase 2	Advanced NSCLC
			Phase 2	Metastatic PDAC
			Phase 2	Advanced solid tumors
